# Supplementary material for: The interactome of intact mitochondria by cross-linking mass spectrometry provides evidence for coexisting respiratory supercomplexes
Source: Mol Cell Proteomics. 2017 Dec 8;17(2):216–32. doi: 10.1074/mcp.RA117.000470 (PMC5795388; doi:10.1074/mcp.RA117.000470)
Supplement: Supplemental Data [file supp_RA117.000470_133922_0_supp_23978_fzffwf.zip › spectra_annotation/mito_DR_spectra_annotation/374-1-3-1-9-1.pdf]

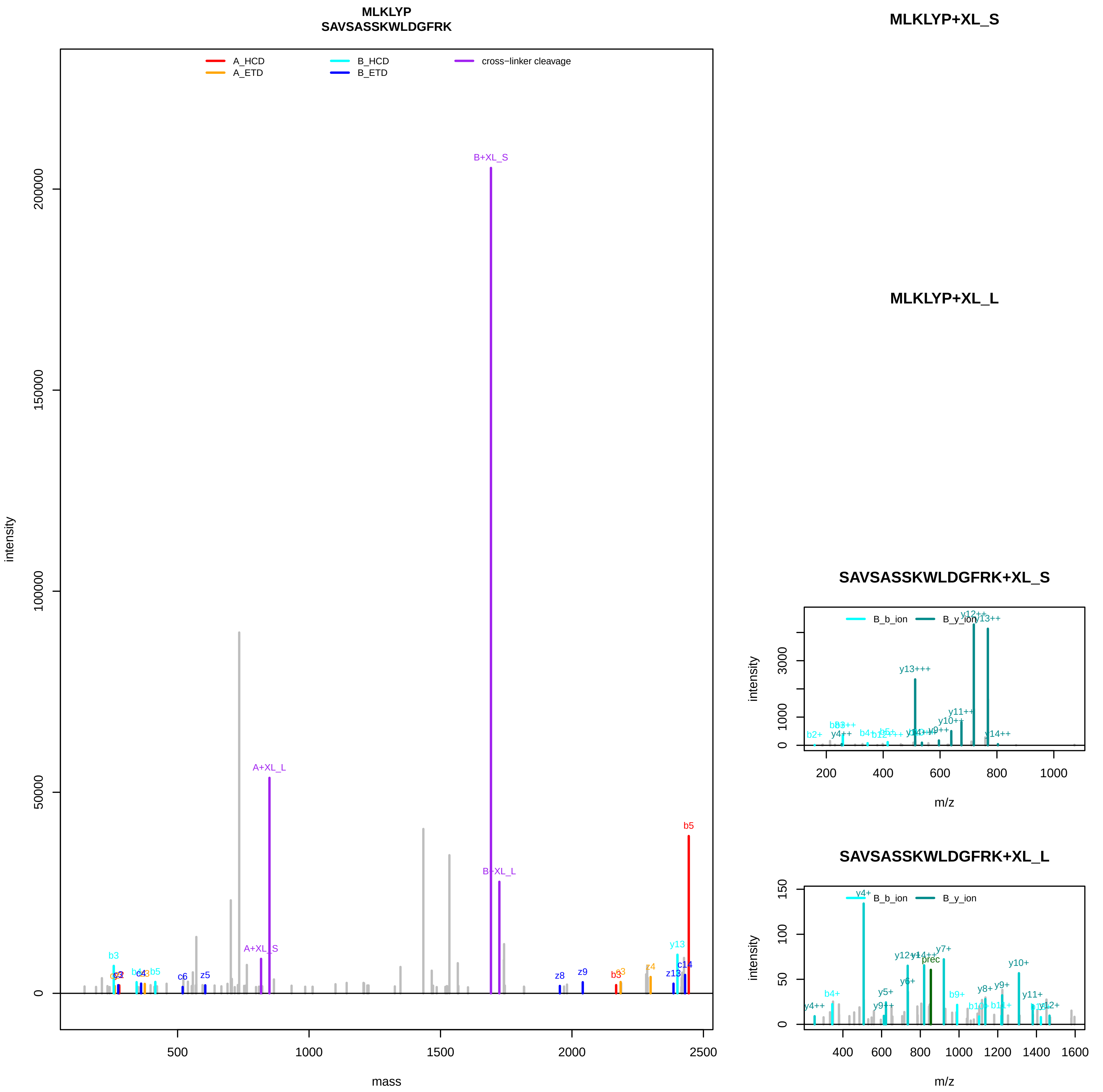
MLKLYP
SAVSASSKWLDGFRK
A_HCD
A_ETD
B_HCD
B_ETD
cross−linker cleavage
B+XL_S
A+XL_L
B+XL_L
A+XL_S
intensity
mass
0
50000
100000
150000
200000
500
1000
1500
2000
2500
b3
b5
c6
z5
z8
z9
z13
y13
c14
MLKLYP+XL_S
MLKLYP+XL_L
SAVSASSKWLDGFRK+XL_S
B_b_ion
B_y_ion
y13+++
y11++
y10++
y14++
m/z
200
400
600
800
1000
0
1000
3000
SAVSASSKWLDGFRK+XL_L
y4+
y4++
b4+
y5+
y6+
y7+
prec
y9+++
b9+
y8+
y9+
y10+
y11+
y12+
0
50
100
150
400
600
800
1000
1200
1400
1600
